# Brown bear and Persian leopard attacks on humans in Iran

**DOI:** 10.1371/journal.pone.0255042

**Published:** 2021-07-22

**Authors:** Jamshid Parchizadeh, Jerrold L. Belant

**Affiliations:** Global Wildlife Conservation Center, College of Environmental Science and Forestry, Department of Environmental and Forest Biology, State University of New York, Syracuse, New York, United States of America; Universita degli Studi di Firenze Dipartimento di Biologia, ITALY

## Abstract

Large carnivore attacks on humans are a serious form of human-wildlife interaction which has increased globally in recent decades. When attacks occur, both humans and large carnivores suffer, highlighting the need to characterize these conflicts toward mitigation of attacks. We investigated brown bear (*Ursus arctos*) and Persian leopard (*Panthera pardus*) attacks on humans across Iran using reports provided by the Government of Iran during 2012–2020. We characterized temporal and spatial patterns of attacks, as well as species-specific attributes. We identified 83 attacks resulting in 77 human injuries and 6 fatalities. Bears were responsible for more attacks (63%) than leopards (37%). Attacks occurred more frequently during defensive reactions by bears and leopards on adult male people while livestock herding during the day in spring and summer. Bears reportedly attacked people more often in western provinces of Iran, while leopards attacked more frequently in northern provinces. We recommend that the Iran Department of the Environment consider implementing a national reporting system to document bear and leopard attacks on people. We further suggest development of national bear and leopard management plans that emphasize mitigating human risk to improve human attitudes toward these carnivore species to facilitate their conservation.

## Introduction

Human populations continue to expand worldwide, resulting in increased encroachment into areas inhabited by large carnivores [1, 2]. A serious form of human-carnivore interaction is attacks on people that can result in human injury or death [3–6]. Further negative attitudes toward carnivores are often reinforced following attacks on people which can have long-term conservation consequences for large carnivore populations [7, 8]. It is therefore important to mitigate carnivore attacks on humans [2].

Potential for human-carnivore interactions including attacks can vary temporally. For example, increased human outdoor activities during spring and summer can increase encounters with carnivores [9, 10]. Prevalence of outdoor activities also varies by time of day, with greater frequency during the day [10, 11]. Human-carnivore interactions also vary by human activity and among human gender or age class. For instance, livestock in many parts of the world more typically are tended by adult males, potentially increasing their exposure to carnivores [9, 12–15]. In Asia, some protected areas are interspersed with crop fields, forest reserves, and human settlements, which can result in frequent carnivore attacks on humans and in many cases, retaliatory killings of carnivores [16–18].

Brown bears (*Ursus arctos*) are estimated to exceed 200,000 individuals worldwide inhabiting North America, Europe, and Asia [19]. However, the status of brown bear populations is poorly known in Asia [20]. Brown bears occur in northern (Alborz Mountains), north-western (Arasbaran Biosphere Reserve), and western (Zagros Mountains) Iran, and the main forms of human-bear conflicts include illegal shooting and stoning, vehicle collision, and habitat degradation or loss [20]. Brown bears are opportunistic omnivores with an amazingly diverse diet and, because of their size and winter hibernation, they must consume large quantities of nutritional food to satisfy their energetic demands [21]. Although bears can alter their behavior to avoid humans [22], complete avoidance is not always possible [23], and bear attacks on people may occur [1, 24]. Most bear attacks on humans in the wild include defensive acts by bears [25, 26]. Defensive reactions in response to sudden encounters with people can occur from female bears with dependent young or bears at a food source (e.g. animal carcass) [27]. Leopards (*Panthera pardus*) have a wide geographic range extending across much of Africa and Asia from the Middle East to the Pacific Ocean [28, 29]. According to unsubstantiated estimates, between 550 and 850 Persian leopards (*P*.*p*. *tulliana* = *P*.*p*. *ciscaucasica* = *P*.*p*. *saxicolor*) occur Iran, specifically in the Alborz and Zagros Mountains, which comprise more than 75% of this subspecies current range [30]. A primary reason for human-leopard conflicts is linked to the extent to which leopards kill livestock and dogs [31]. Leopards also attack humans [32, 33] in defensive acts [34–36]. For example, leopards attack humans as a defensive reaction when wounded, trapped, or cornered, with mauling much more common than death [35].

Iran is experiencing rapid human population growth and urbanization, resulting in a profound socio-demographic change from a primarily agricultural to developing nation [37]. This socio-demographic shift has resulted in substantial land use change and led to increased human-wildlife conflicts in Iran [38, 39]. Specifically, there has been an increase in severity of human-carnivore conflicts in Iran in the last decade involving brown bear and Persian leopard [31, 40–42], and more detailed understanding of conflict patterns to potentially reduce these attacks is warranted. Our objective was to characterize temporal and spatial patterns of brown bear and Persian leopard attacks on people in Iran. We predicted that bears and leopards would be involved more often in defensive attacks on humans, and that these attacks would occur more frequently on adult male people while livestock herding during the day in spring and summer.

## Materials and methods

### Study area

**Fig 1:**
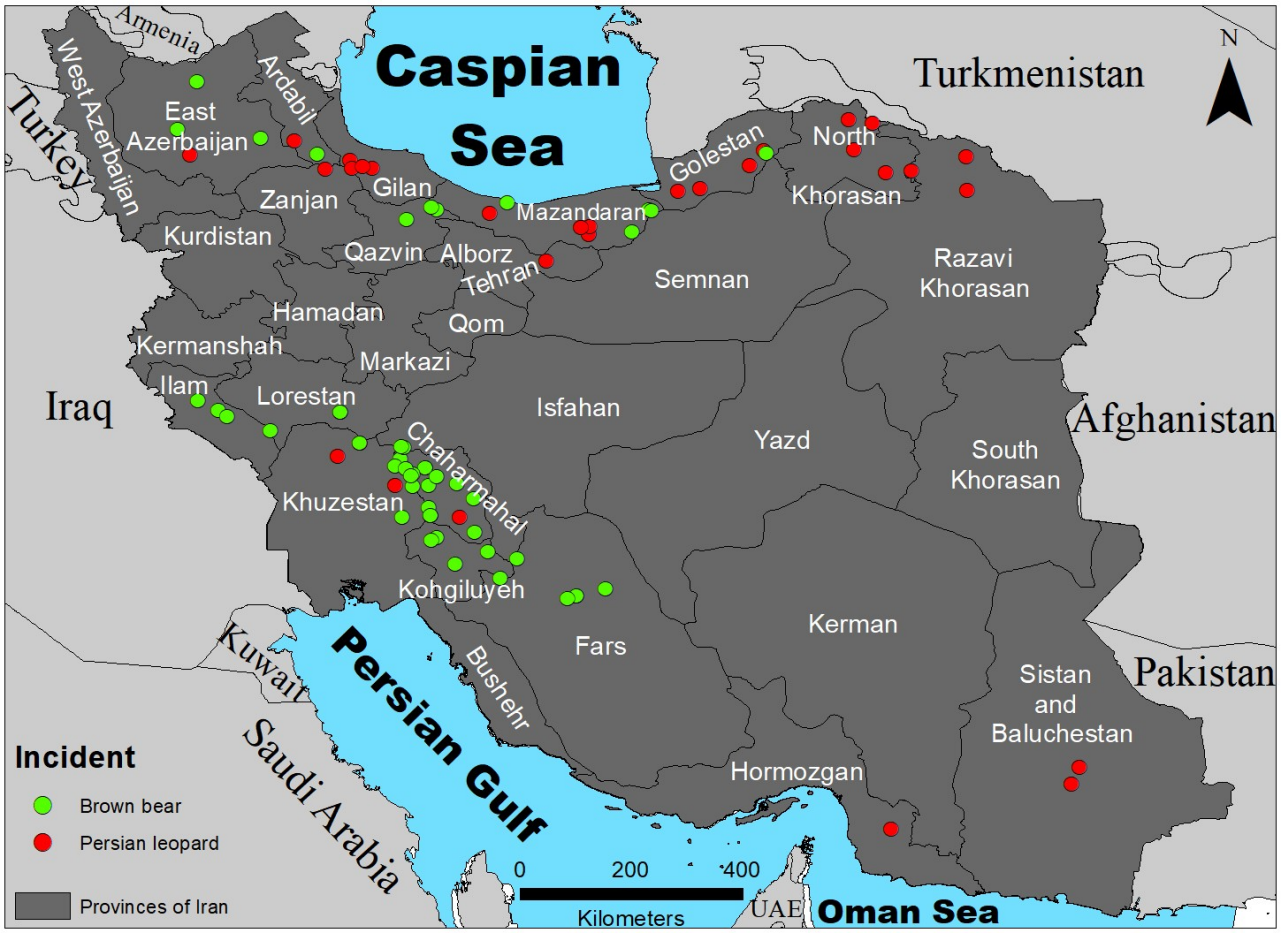
Locations of human-brown bear (*Ursus arctos*) and human-Persian leopard (*Panthera pardus*) incidents, Iran, 2012–2020.

Iran comprises 1,648,195 km^2^ in southwestern Asia (25–40° N, 44–64° E), containing 31 provinces (Fig 1). Major mountain ranges include the Alborz and Zagros which occur across the northern and western parts of Iran, respectively [43]. Overall climate is typically arid or semi-arid, except for coastal areas in the north and parts of western Iran; annual rainfall decreases from 1800 mm in the north to < 100 mm in central arid regions [44]. The climate is continental with hot, dry summers and cold winters; mean monthly temperatures range from 6.5°C in January to 31°C in July [45].

### Data collection

We conducted a Farsi gray literature search for brown bear (equivalent Farsi words are خرس قهوه ای and خرس) and Persian leopard (پلنگ ایرانی and پلنگ) attacks on humans in Iran using the Google web search engine to access and compile all relevant material confirmed, published, and disseminated by responsible bodies of the Government of Iran including the Department of the Environment (DOE), Veterinary Organization, Provincial governorships, hospitals, and Universities of Medical Sciences, during 2012–2020. We used the search terms: “common species name” + “attack (حمله)” + “human/people (انسان)” for each species.

We defined casualties as bear and leopard attacks causing human injury or fatality and incidents as human-bear and human-leopard encounters that led to ≥1 casualty; therefore, the total number of casualties was greater than the total number of incidents [24]. We included only incidents that resulted in human casualty.

We compiled several variables for each casualty or incident when available including target species (i.e. brown bear or leopard) involved, number of people involved, and outcome of the incident (injury or fatality). We categorized primary activity of humans before incidents as indoor (i.e. activity in the yard including resting and sleeping), job-related (i.e. conservation work), outdoor recreation (e.g. camping, hiking, biking, rock climbing, and gathering wild resources), livestock herding, or other outdoor. We classified bear and leopard attack motivations as predatory to livestock (e.g. when humans were attacked while protecting their livestock from bears or leopards) or defensive act by animal (e.g. when bears or leopards were defending their young or were surprised, harassed, or provoked by humans). We categorized age of humans as child (0–9 years old), adolescent (10–18 years old), and adult (> 18 years old). We identified human gender (male or female) involved in the incident. We classified group size of people as one, two, or three. We recorded time of day as day (sunrise to sunset), night (sunset to sunrise), or unknown and month and year. When possible, we estimated the geographic location of each incident (typically within 5 kilometers) and plotted them using ArcGIS 10.3 (ESRI, Redlands, CA) at the province level.

We identified multiple sources for some incidents and selected up to two sources that contained the greatest amount of information. We then reviewed each incident and compared attributes to prevent duplicate records in our dataset.

We used two-way χ^2^ tests in program R [46] to compare differences in the frequency of bear and leopard incidents by time of day; month; attack motivation of animals; and primary activity, gender, and age class of people. We used α of ≤ 0.05 to denote statistical significance.

## Results

We identified 83 reported incidents involving 90 human casualties. Bears were responsible for more incidents (63%; *n* = 52) than leopards (37%; *n* = 31). Bear incidents caused 90% (*n* = 47) human injuries and 10% (*n* = 5) fatalities, whereas leopard incidents resulted in 97% (*n* = 30) injuries and 3% (*n* = 1) fatalities.

**Fig 2:**
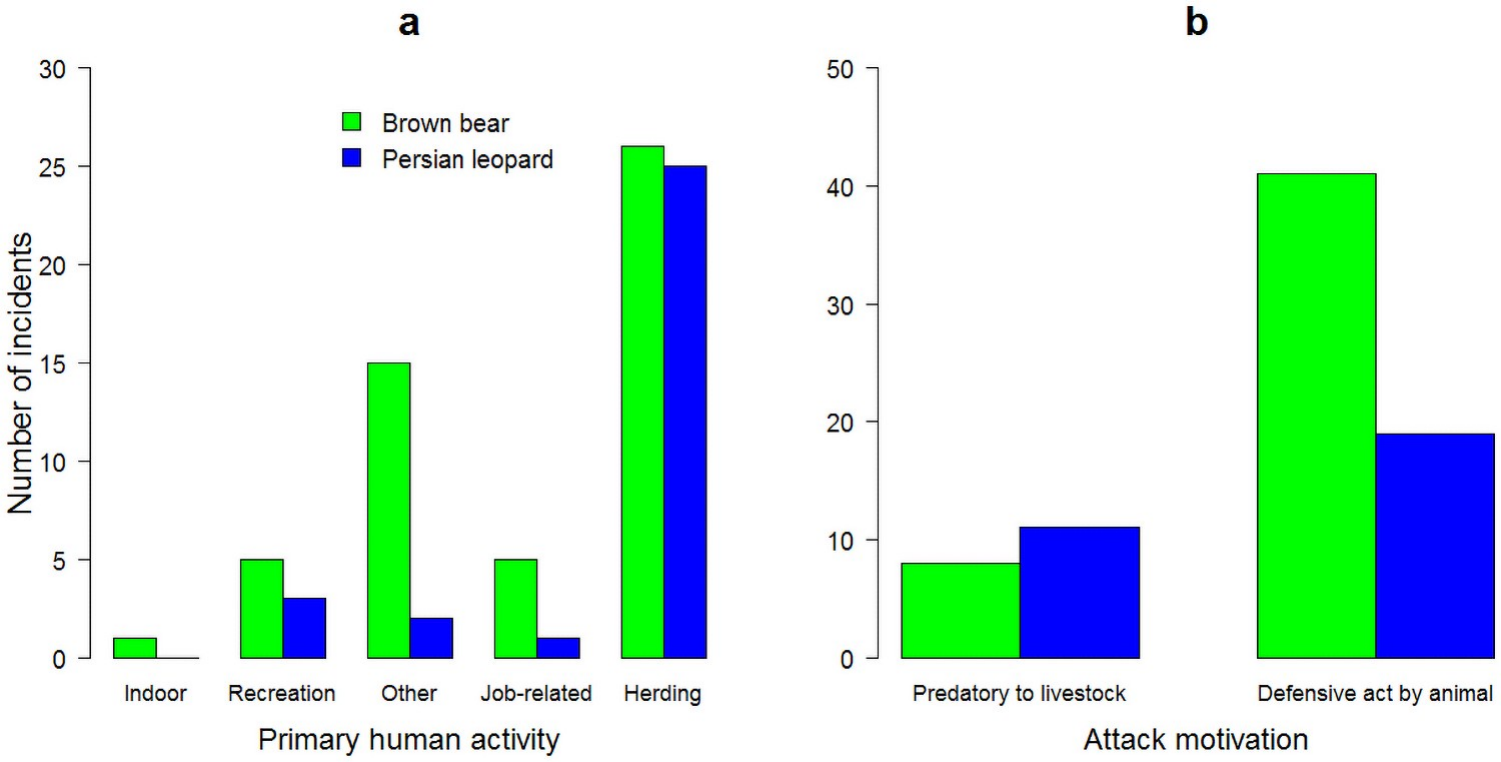
Number of human-brown bear (*Ursus arctos*) and human-Persian leopard (*Panthera pardus*) incidents by type of primary human activity (a) and attack motivation (b), Iran, 2012–2020.

Most incidents occurred with people were herding livestock (61%; χ^2^ = 9.4, d.f. = 4, *p* = 0.05) than other activities (Fig 2a). Bears attacked humans more frequently while livestock herding (50% of incidents), followed by people engaged in other outdoor activities (29% of incidents). Similarly, leopards attacked people more often while livestock herding (81% of incidents), followed by people engaged in outdoor recreation (10% of incidents). Furthermore, bears and leopards were involved more frequently in defensive acts on humans (72% of all incidents; χ^2^ = 4.2, d.f. = 1, *p* = 0.04). Bears (79% of bear incidents) and leopards (61% of leopard incidents) were defensive reactions to human activities (Fig 2b).

**Fig 3:**
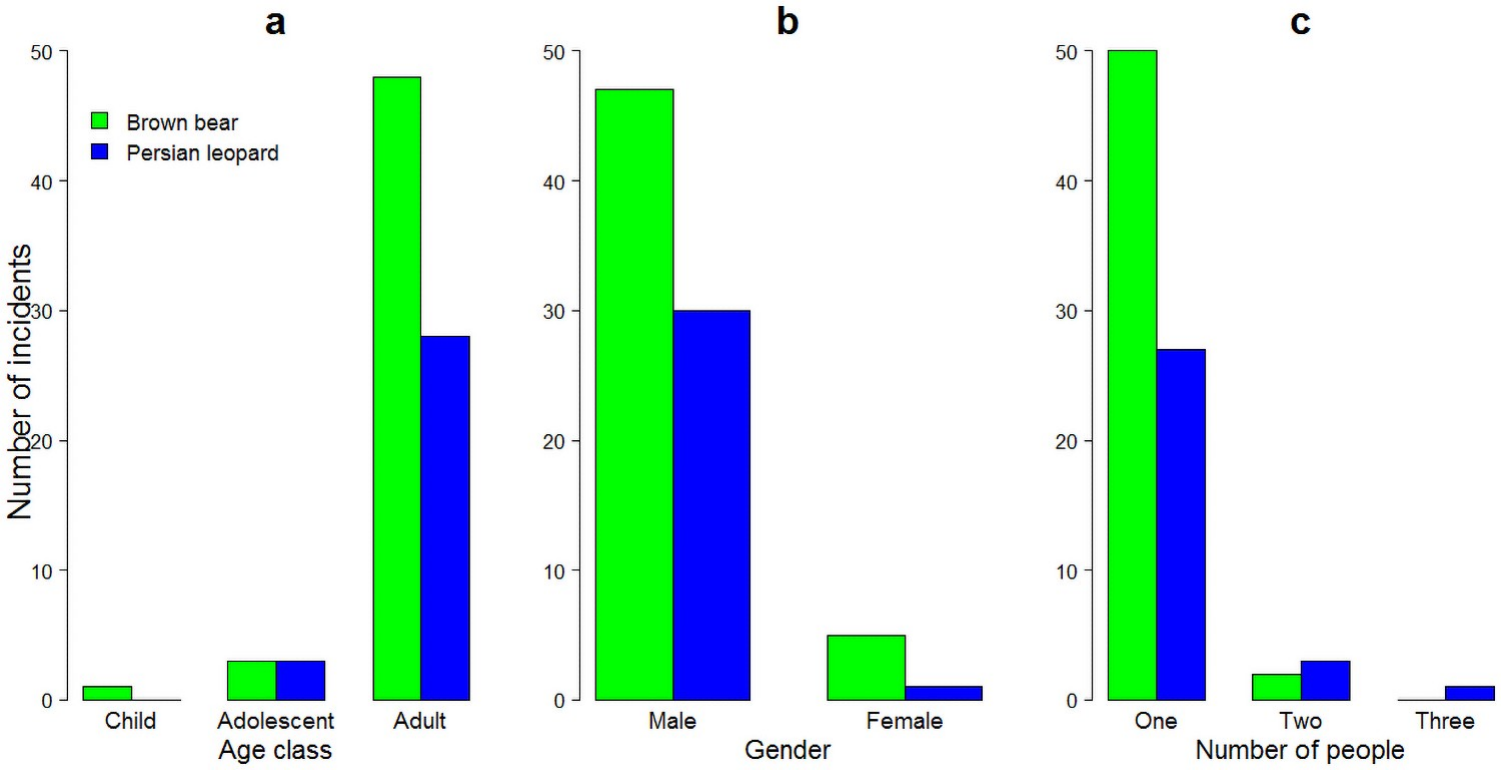
Number of human-brown bear (*Ursus arctos*) and human-Persian leopard (*Panthera pardus*) incidents by age class (a), gender (b), group size (c) of people, Iran, 2012–2020.

Adults were attacked more often by bears and leopards (92% of all incidents; χ^2^ = 1, d.f. = 2, *p* = 0.60) than were adolescents and children. More adults were attacked by bears (92% of bear incidents) and leopards (90% of leopard incidents; Fig 3a), and male people were involved in incidents thirteen times more frequently than females (93% of all incidents; χ^2^ = 1.2, d.f. = 1, *p* = 0.28). Bears (90% of bear incidents) and leopards (97% of leopard incidents) attacked considerably more males (Fig 3b). Furthermore, in 93% (*n* = 77) of incidents the person attacked was alone (Fig 3c).

**Fig 4:**
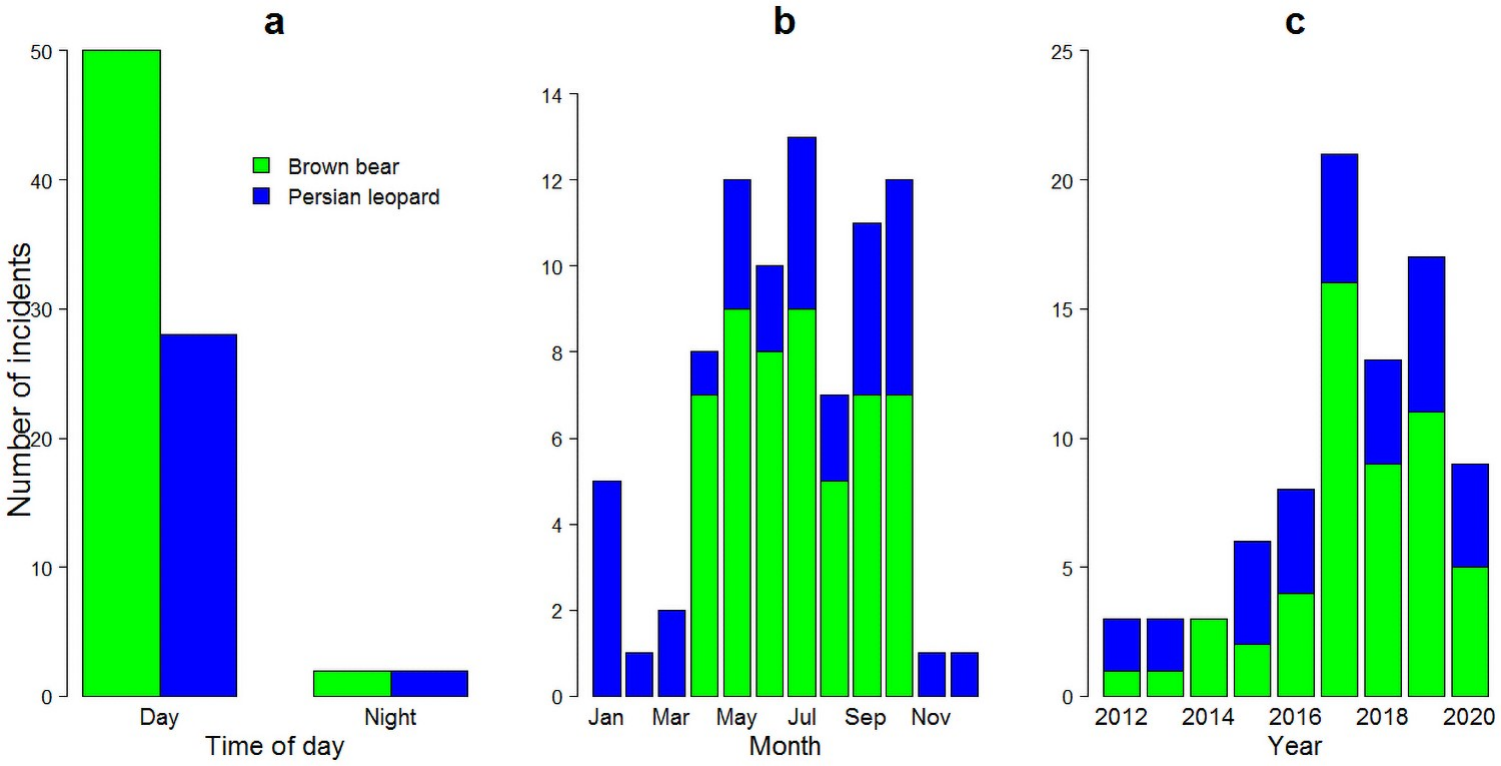
Number of human-brown bear (*Ursus arctos*) and human-Persian leopard (*Panthera pardus*) incidents by time of day (a), month (b), and year (c), Iran, 2012–2020.

Incidents during the day occurred about twenty times more often than at night (94% of all incidents; χ^2^ = 0.3, d.f. = 1, *p* = 0.57). Specifically, bears (96% of bear incidents) and leopards (90% of leopard incidents) attacked more during the day (Fig 4a). Additionally, humans were attacked more often during spring–summer (73% of all incidents; χ^2^ = 21.5, d.f. = 11, *p* = 0.03); leopard incidents occurred during every month of the year, while bear incidents occurred during April–October (Fig 4b). The annual number of reported incidents doubled from 2012 to 2020 with the greatest numbers in 2017 (*n* = 21), 2019 (*n* = 17), and 2018 (*n* = 13; Fig 4c).

Most bear incidents (573%; *n* = 38) occurred in western provinces, particularly Chaharmahal and Bakhtiari (37%), followed by Khuzestan (21%), and Kohgiluyeh and Boyer-Ahmad (13%). Most leopard incidents (61%; *n* = 19) occurred in northern provinces with Mazandaran and North Khorasan having 26% each, followed by Gilan and Golestan having 21% each (Fig 1).

## Discussion

Our predictions of patterns influencing brown bear and Persian leopard incidents in Iran were largely supported. Our results suggest that incidents occurred more often from defensive reactions by bears and leopards and attacks most commonly involved adult male people while livestock herding during the day in spring and summer.

Most bear and leopard incidents occurred while people herded livestock, and these incidents were mostly defensive reactions by bears and leopards, as found previously [36, 47–49]. This outcome was probably linked to the animals being disturbed or surprised due to human presence in bear and leopard habitats. For example, animal husbandry practices which require travel into bear and leopard habitats can increase potential for incidents [49]. Iranian herders hold official grazing permits which specify the locations and periods for grazing, but many herders allow livestock to overgraze pastures and then move livestock into protected areas where bears and leopards occur [42, 50]. Although challenging, encouraging shepherds to use alternative animal husbandry practices (e.g. night penning) instead of traditional practices (e.g. free-range grazing) to lessen reliance on pastures [50] may reduce such encounters.

Bears and leopards attacked more adults than adolescents and children, which supports previous studies [10, 24, 29, 51]. This observed pattern is probably linked to increased numbers of adults involving in professional and recreation outdoor activities, resulting in greater levels of human disturbance and aggressive responses from bears and leopards [1, 23, 24, 52, 53].

Most reported incidents by bears and leopards involved male people and occurred during the day, consistent with previous studies [24, 29, 51]. This outcome is likely related to religion and tradition in Iran where culturally, 98% of people are Muslim [54] and within Iranian culture, males perform most outdoor activities [55], resulting in more exposure to bear and leopard incidents. In addition, the increased number of incidents during the day might be due to greater human activity [10, 53], which would increase probability of encounters with bears and leopards.

Observed seasonal patterns in bear incidents are supported by previous studies [10]. Our results indicated that bear incidents increased slightly during May-July, suggesting that both bears and humans were most active during this time. Longer day length can result in increased bear activity [56] which makes bears more likely to encounter humans. Additionally, the lack of reported bear incidents during winter reflects seasonal denning behavior by bears [1]. Furthermore, lush vegetation attracts more cattle for free-range grazing and longer day length in spring and summer allows cattle to move deeper into leopard habitats [57], which in turn may lead to increased human-leopard incidents.

We demonstrated an increasing number of bear and leopard incidents in Iran during 2012–2020. This trend may be linked to increased reporting of incidents due to improved electronic communications (e.g. internet) by the Government of Iran. However, many incidents may remain unreported due to the lack of a standardized and easy to use reporting system which individuals or communities could adopt to report incidents [49]. A standardized reporting system also would reduce potential for duplicate reporting and provide a mechanism for DOE experts to contact individuals for validation of incidents.

Brown bear incidents occurred more frequently in western provinces which contain the Zagros Mountains. Brown bears are more common in western provinces of Iran [58]. Furthermore, about 36% of Iran’s rural and nomadic families as well as 52% of all livestock occur in the Zagros region [59]. Consequently, the primary occupation of people is livestock herding [60] which overlaps with brown bear range and may result in increased incidents [40]. In contrast, most leopard incidents occurred in the Alborz Mountains (northern provinces), perhaps due to the leopards’ greater occurrence in this region [30, 42]. Substantial and widespread land use changes have occurred in northern provinces of Iran recently [61–63]. Increased habitat encroachment by people can reduce suitable leopard habitat and contribute to increased human-leopard incidents [35, 64].

The Iranian DOE issues licenses annually to foreign and Iranian hunters to harvest wild ungulates including urial wild sheep *Ovis vignei*, bezoar goat *Capra aegagrus*, and red deer *Cervus elaphus*, primary prey of carnivores [65, 66]. These ungulates are also subject to poaching [67]. Increased human encroachment can result in wild prey depletion [68–71], and therefore bears and leopards may switch from wild to domestic prey and consume other anthropogenic food resources [7, 69, 72–77], which in turn can further increase bear and leopard incidents [71]. Bears and leopards can be attracted to livestock carcasses not properly disposed of near populated areas and also to other anthropogenic-derived food and food waste that are improperly stored [10, 27], an observed pattern for wolves in western Iran which resulted in increased wolf incidents [78, 79]. Thus, removing these attractive foods from human-occupied areas could reduce bear and leopard use of these areas and avoid potential incidents [10]. For instance, during Christian fasting periods when food waste was limited, spotted hyenas (*Crocuta crocuta*) increased predation of domestic donkeys instead of scavenging food waste [80].

Our study may have potential inconsistencies and reporting bias due to sensitive information regarding details of bear and leopard incidents with humans. The site of large carnivore incidents should be described and analyzed with similar criteria as for human crime scenes, to ensure detailed documentation occurs [15]. We recognize that face-to-face interviews with affected parties and witnesses would have improved the quality of our data. Nonetheless, we expect that our use of official Iranian government reports decreased this potential bias.

When bear and leopard incidents occur, both people and animals suffer [1, 81] with negative attitudes towards these species increasing [6, 7, 82]. It is therefore crucial to design and conduct effective strategies aimed at human safety and brown bear and leopard conservation. Our study can help mitigate bear and leopard incidents in Iran by informing wildlife managers and the public about circumstances surrounding such incidents, and measures to reduce their occurrence.

## Supporting information

S1 Table Number of human-brown bear (*Ursus arctos*) and human-Persian leopard (*Panthera pardus*) incidents by province in Iran during 2012–2020, and relevant data sources. (XLSX)
